# Comparative Genomics of the First and Complete Genome of “*Actinobacillus porcitonsillarum*” Supports the Novel Species Hypothesis

**DOI:** 10.1155/2018/5261719

**Published:** 2018-09-30

**Authors:** Valentina Donà, Vincent Perreten

**Affiliations:** Institute of Veterinary Bacteriology, Vetsuisse Faculty, University of Bern, Bern, Switzerland

## Abstract

“*Actinobacillus porcitonsillarum*” is considered a nonpathogenic member of the *Pasteurellaceae* family, which phenotypically resembles the pathogen *Actinobacillus pleuropneumoniae*. Previous studies suggested that “*A. porcitonsillarum*” may represent a new species closely related to *Actinobacillus minor*, yet no full genome has been sequenced so far. We implemented the Oxford Nanopore and Illumina sequencing technologies to obtain the highly accurate and complete genome sequence of the “*A. porcitonsillarum*” strain 9953L55. After validating our *de novo* assembly strategy by comparing the *A. pleuropneumoniae* S4074^T^ genome sequence obtained by Oxford Nanopore Technology combined with Illumina reads with a PacBio-sequenced S4074^T^ genome from the NCBI database, we performed comparative analyses of the 9953L55 genome with the *A. minor *type strain NM305^T^, *A. minor* strain 202, and *A. pleuropneumoniae* S4074^T^. The 2,263,191 bp circular genome of 9953L55 consisted of 2168 and 2033 predicted genes and proteins, respectively. The lipopolysaccharide cluster resembled the genetic organization of *A. pleuropneumoniae* serotypes 1, 9, and 11, possibly explaining the positive reactions observed previously in serotyping tests. In contrast to NM305^T^, we confirmed the presence of a complete *apxIICABD* operon in 9953L55 and 202 accounting for their hemolytic phenotype and Christie-Atkins-Munch-Petersen (CAMP) reaction positivity. Orthologous gene cluster analysis provided insight into the differential ability of strains of the *A. minor*/“*porcitonsillarum*” complex and *A. pleuropneumoniae* to ferment lactose, raffinose, trehalose, and mannitol. The four strains showed distinct and shared transposable elements, CRISPR/Cas systems, and integrated prophages. Genome comparisons based on average nucleotide identity and *in silico* DNA-DNA hybridization confirmed the close relationship among strains belonging to the *A. minor*/“*porcitonsillarum*” complex compared to other *Actinobacillus* spp., but also suggested that 9953L55 and 202 belong to the same novel species closely related to *A. minor*, namely, “*A. porcitonsillarum*.” Recognition of the taxon as a separate species would improve diagnostics and control strategies of pig pleuropneumonia.

## 1. Introduction

“*Actinobacillus porcitonsillarum*” is a Gram-negative rod belonging to the *Pasteurellaceae* family, which is regularly isolated from the tonsils of healthy pigs and phenotypically resembles *Actinobacillus pleuropneumoniae*, the causative agent of porcine pleuropneumonia, which is associated with high economic burdens in the pig industry worldwide [1, 2]. “*A. porcitonsillarum*” mimics the major antigenic factors of *A. pleuropneumoniae* causing cross-reactivity in serological tests [1], which negatively affects serological diagnosis of *A. pleuropneumoniae*, potentially leading to the unnecessary depopulation and/or antimicrobial treatment of pig herds.

The “*A. porcitonsillarum*” strain 9953L55 (CCUG 46996) was firstly isolated from the tonsils of a healthy pig belonging to a high-health status herd considered to be free from *A. pleuropneumoniae*, in which regular serological testing suddenly evidenced a low number of pigs showing weak positive reactions for *A. pleuropneumoniae* serogroups 1, 9, and 11 [3]. Subsequent phenotypic and biochemical analyses indicated that this strain appeared to be identical to *A. pleuropneumoniae*, including the hemolytic growth on blood agar plates and the Christie-Atkins-Munch-Petersen (CAMP) activity, i.e., a cohemolytic effect observed on blood agar plates in the presence of a sphingomyelinase (*β*-hemolysin)-producing *Staphylococcus aureus*, but with the exception that it did not ferment mannitol [1]. Serotyping by three different methods showed a positive reaction with antiserum raised against serotype 1 *A. pleuropneumoniae* S4074^T^ [1]. Nevertheless, three *A. pleuropneumoniae*-specific PCRs were negative, indicating also the absence of the *apxIV* gene, which was previously proven to be species-specific for *A. pleuropneumoniae* [1, 4]. Toxin gene typing PCR for the major RTX toxins (ApxI, ApxII, and ApxIII) additionally revealed that *apxII*, but not *apxI* or *apxIII* genes were present [1].

Phylogenetic analysis of the 16S rRNA gene sequence indicated that “*A. porcitonsillarum*” was most closely related to *Actinobacillus minor* strain 202 (formerly named “*Haemophilus* strain 202”, but subsequently classified as a borderline *A. minor* strain [5]), and to the *A. minor* type strain NM305^T^, although it distinguished itself phenotypically from the latter by the hemolysis and CAMP activity [1]. Interestingly, a later study provided evidence that *A. minor* 202 also produced the ApxII toxin and appeared to be genetically rather more related to “*A. porcitonsillarum*” than to *A. minor* NM305^T^ [6].

Despite these previous observations suggesting that “*A. porcitonsillarum*” may represent a new species, it has not been recognized as a distinct species so far, mainly due to the absence of sufficient phenotypic markers to distinguish it from *A. minor* [7]. However, a clear differentiation of the commensal “*A. porcitonsillarum*” from the pathogen *A. pleuropneumoniae* would be essential in diagnostics and, particularly, in eradication programs.

To corroborate these previous observations at a genomic level, we used the Oxford Nanopore and Illumina sequencing technologies to sequence the “*A. porcitonsillarum*” strain 9953L55, which was proposed as a type strain for “*A. porcitonsillarum*.” After the validation of our *de novo* assembly approach by obtaining the genome of the *A*. *pleuropneumoniae* strain S4074^T^ with the Oxford Nanopore Technology combined with Illumina reads and comparing it with the PacBio-sequenced genome of the same strain found in the NCBI database, we implemented this method to obtain the highly accurate circular genome sequence of strain 9953L55, which was further used for comparative analyses with the genome sequences of *A. minor* 202, *A. minor* NM305^T^, and *A*. *pleuropneumoniae* S4074^T^.

## 2. Materials and Methods

### 2.1. Bacterial Strains, Growth Conditions, and Sugar Fermentation Test

“*A. porcitonsillarum*” 9953L55 (CCUG 46996), *A. minor* NM305^T^ (CCUG 38923^T^), and *A. pleuropneumoniae* S4074^T^ (ATCC 27088^T^) were grown on chocolate agar plates supplemented with Polyvitex (BioMérieux) at 37°C with 5% CO_2_. Lactose, raffinose, and trehalose fermentation was assessed, using S4074^T^ and NM305^T^ as control strains, in PPLO broth (Difco) supplemented with 40 *μ*g/ml NAD, as described previously [1, 8].

### 2.2. DNA Isolation and Sequencing

DNA was isolated with a modified phenol/chloroform extraction method, treated for 30 min with 0.5 *μ*l RNase (20 mg/ml) (Qiagen), and purified with 0.8X Agencourt AMPure beads (Beckman Coulter) [9]. The purified DNA was subsequently sheared to 8–10 kb fragments with a g-TUBE (Covaris), and library preparation was performed with the SQK-LSK108 1D ligation sequencing kit (Oxford Nanopore), as per the manufacturer's instructions. The sequencing library was sequenced on a R9.4 SpotON flow cell (Oxford Nanopore) with the MinION Mk 1B sequencing device (Oxford Nanopore) for 24 hours. In parallel, the DNA was also submitted to GATC, Constance, Germany, for 2 × 150 paired-end sequencing on an Illumina HiSeq (Illumina) platform.

### 2.3. Genome Assembly

Base calling and quality filtering of the Oxford Nanopore Technology (ONT) reads were performed with Albacore v2.0.1. Pairing, trimming, and quality filtering of the Illumina reads were performed with Trimmomatic v0.33. ONT reads were assembled with Canu v1.3 with default parameters and the option corOutCoverage = 100 [10]. Paired-end Illumina reads were mapped to the Canu-generated scaffold with BWA-MEM v0.7.13 and polished with Pilon 1.22 twice [11]. A third mapping of the Illumina reads was performed with BWA-MEM for the final inspection and curation of the polished sequence with the Geneious software v10.2.3 (Biomatters). In case of repetitive regions leading to unbalanced (low) read coverage, these regions were extracted to locally repeat read mapping with BWA-MEM. The final circular genome sequences of strains 9953L55 and S4074^T^ were first annotated with Prokka v1.12 for primary sequence analysis and subsequently with the NCBI prokaryotic genome annotation pipeline [12]. Paired-end Illumina reads were used to run plasmidSPAdes v3.9.0 with default parameters [13].

### 2.4. Genome Analysis and Comparison

The whole-genome shotgun and complete genome sequences, which were retrieved from the NCBI database for the genome comparisons, are deposited under the following GenBank accession numbers: *A. minor* NM305^T^ (ACQL01000001-ACQL01000197), *A. minor* 202 (ACFT01000001-ACFT01000154), *A. pleuropneumoniae* S4074^T^ (PacBio, CP029003; Roche 454, ADOD01000001-ADOD01000044), *A. equuli* 19392^T^ (CP007715), *A. succinogenes* 130Z^T^ (NC_009655), *A. suis* ATCC 33415^T^ (NZ_CP009159), *A. ureae* ATCC 25976^T^ (AEVG01000001-AEVG01000183), *A. capsulatus* DSM 19761^T^ (ARFN01000001-ARFN01000049), and *A. seminis* ATCC 15768^T^ (NLFK01000001-NLFK01000022). Genome alignments were performed with progressiveMauve v2.3.1 [14]. OrthoVenn was used to identify orthologous genes [15]. Online available platforms were used to characterize the presence of known resistance genes (ResFinder) [16], plasmids (PlasmidFinder) [17], insertion sequences (IS, ISfinder) [18], clustered regularly interspaced short palindromic repeat (CRISPR) arrays and CRISPR-associated gene (CRISPR/Cas) systems (CRISPRone) [19], and phage sequences (PHASTER) [20]. The circular map of 9953L55 including the BLAST-based comparison with the genome sequences of *A. minor* NM305^T^, *A. minor* 202, and *A. pleuropneumoniae* S4074^T^ was generated with the BLAST Ring Image Generator (BRIG) [21]. Comparisons of average nucleotide identity (ANI) based on BLAST and MUMmer pairwise sequence alignments (ANIb and ANIm, respectively) were obtained with JSpeciesWS [21, 22]. The distance matrix representing the ANI divergence (defined as 100% − ANI) was used to compute a complete linkage hierarchical clustering with the hclust function in R v3.0.1, as done previously [23]. *In silico* DNA-DNA hybridization (*is*DDH) based on genome BLAST distance phylogeny was performed with GGDC 2.1 [24]. Only results based on formula 2 were used for analysis, since it estimates *is*DDH values independently of genome length and is therefore recommended for incomplete genomes [25, 26].

### 2.5. Nucleotide Sequence GenBank Accession Number

The complete nucleotide sequences of the “*A. porcitonsillarum*” strain 9953L55 and of the *A. pleuropneumoniae* strain S4074^T^ were deposited in DDBJ/EMBL/GenBank under the accession numbers CP029206 and CP030753, respectively.

## 3. Results and Discussion

After base calling and quality filtering, 580,932 1D pass ONT reads corresponding to 4.08 Gbp (>1800X coverage) and 10,478,015 paired-end Illumina reads were obtained for the *A. pleuropneumoniae* strain S4074^T^. Assembly of the ONT reads generated a single 2.32 Mbp contig with overlapping ends, which was circularized and polished with paired-end Illumina reads.

**Figure 1:**
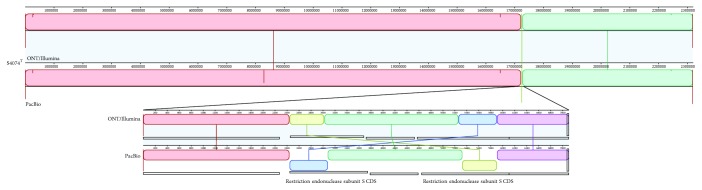
MAUVE alignment of the genome sequence of *A. pleuropneumoniae* S4074^T^ obtained by Oxford Nanopore Technology (ONT)/Illumina (top) and PacBio (bottom) sequencing. The same color boxes, i.e., locally collinear blocks (LCB), represent homologous regions of sequence without rearrangement. The inset underneath magnifies the only rearrangement found between the two sequences.

Alignment of the obtained S4074^T^ genome with the complete genome sequence of the same strain previously sequenced with PacBio technology, which we retrieved from the NCBI database (accession number CP029003), indicated a very high sequence homology (Figure 1). Only one rearrangement was identified between the two genome sequences, which mapped to the 5′-end region of two genes in opposite orientation both encoding a restriction endonuclease subunit S (Figure 1). High sequence divergence was observed solely in a 5 kbp region comprising 5 genes encoding a D-alanine–D-alanine ligase, cell division proteins (*ftsQ*, *ftsA*, and *ftsZ*), and the UDP-3-O-[3-hydroxymyristoyl] N-acetylglucosamine deacetylase (*lpxC*). All 5 genes are annotated as frameshifted and contain internal stop codons in the PacBio-generated S4074^T^ genome sequence. In contrast, all genes were intact in the genome obtained by ONT/Illumina sequencing, and a comparison with a previous whole-genome shotgun assembly of the same strain obtained by Roche 454 sequencing technology, which we retrieved from the NCBI database (accession number ADOD01000001-ADOD01000044), showed 100% identity. A 5 bp indel was found in a gene encoding a methyltransferase annotated as incomplete in the PacBio-generated genome sequence, restoring the completeness of the gene in the genome obtained by ONT/Illumina sequencing. Only 2 additional single nucleotide polymorphisms (SNPs) and one indel (in an intergenic region) were identified between the two genomes.

Taken together, these results confirmed that our approach, i.e., *de novo* assembly of ONT reads combined with Illumina polishing, can be successfully applied to generate a complete and highly accurate bacterial genome sequence. Therefore, this strategy was further used to obtain the full genome sequence of the “*A. porcitonsillarum*” strain 9953L55.

After base calling and quality filtering, 721,267 1D pass ONT reads corresponding to 4.82 Gbp (>2000X coverage) and 5,367,150 paired-end Illumina reads were obtained for strain 9953L55. Assembly of the ONT reads generated a single 2.26 Mbp contig with overlapping ends, which was circularized and polished with paired-end Illumina reads to obtain the complete circular sequence of the “*A. porcitonsillarum*” 9953L55 chromosome, as done for S4074^T^. *In silico* analysis with PlasmidFinder and plasmidSPAdes using paired-end Illumina reads suggested the absence of plasmids.

**Figure 2:**
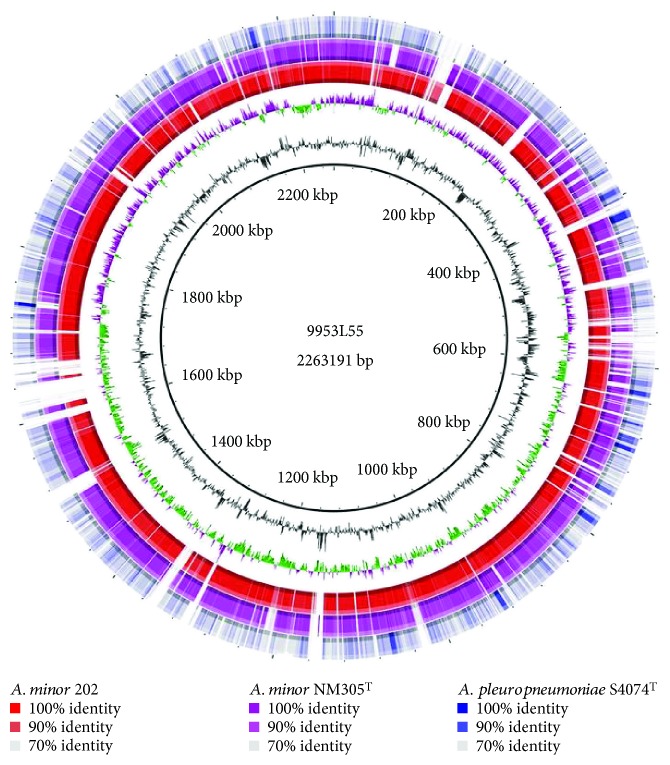
Circular map of the “*A. porcitonsillarum*” strain 9953L55. The scale ring shows the coordinates in kilobase pairs. The second ring represents the average GC content. The third ring represents the GC skew. The colored outer rings display regions of homology based on BLASTn. First outer ring (red): *A. minor* 202, second outer ring (pink): *A. minor* NM305^T^, and third outer ring (blue): *A. pleuropneumoniae* S4074^T^.

The circular genome of 9953L55 consisted of 2,263,191 bp with an average 39.7% GC content and displayed, as expected, a high nucleotide sequence similarity with the genome sequences of *A. minor* NM305^T^ and 202, while *A. pleuropneumoniae* S4074^T^ was more dissimilar (Figure 2). In total, 2168 genes, including six copies of the *rrn* operon encoding the 16S, 23S, and 5S rRNA, as well as 2087 CDS, of which 2033 encode proteins and 54 are pseudogenes, were predicted.

**Figure 3:**
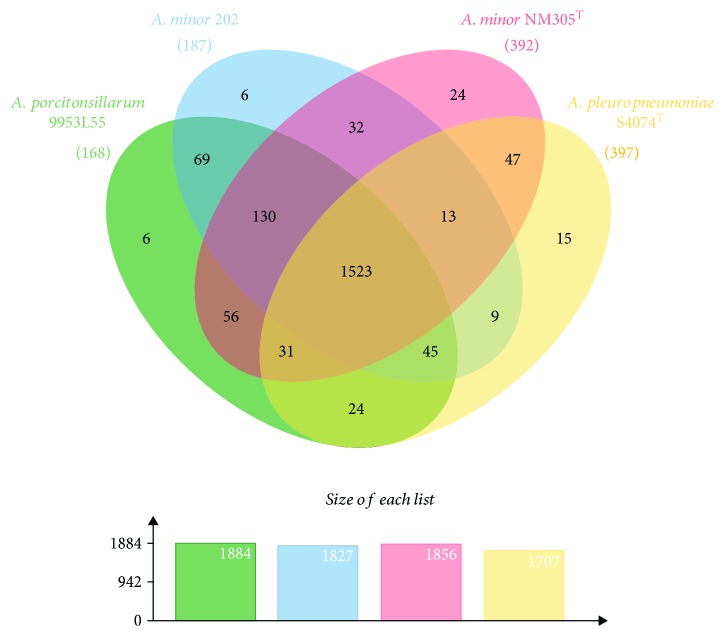
Venn diagram depicting clusters of orthologous genes (COGs) in “*A. porcitonsillarum*” 9953L55, *A. minor* 202, *A. minor* NM305^T^, and *A. pleuropneumoniae* S4074^T^. The number of singletons for each strain is shown in brackets. The total number of COGs for each strain is displayed in the graph underneath.

Analysis with OrthoVenn showed that the four strains display 1523 common clusters of orthologous genes (COGs), of which 1507 were single-copy clusters, indicating few duplication events before speciation (Figure 3). The two *A. minor* strains and “*A. porcitonsillarum*” 9953L55 shared 130 additional COGs, reflecting their closer phylogenetic relationship compared to *A. pleuropneumoniae*, as suggested previously [1]. Interestingly, most of the COGs present only in 9953L55 and S4074^T^ were genes belonging to the lipopolysaccharide (LPS) cluster, which was located (as in the other three strains) between the *erpA* and *rpsU* genes and closely resembled the genetic organization of the *A. pleuropneumoniae* serotype 1, 9, and 11 LPS cluster. This may explain the previously observed cross-reactivity with antiserum against S4074^T^ and, particularly, the positive reaction in the dot-ELISA test with a monoclonal antibody recognizing a common *O*-chain LPS epitope of *A. pleuropneumoniae* serotypes 1, 9, and 11 [1].

The further analysis of the COGs shed some light on the different phenotypes observed in the biochemical tests [1, 5, 27].

RTX toxins (ApxI, ApxII, and ApxIII) in *A. pleuropneumoniae* are responsible for its hemolytic activity and CAMP positivity [28, 29]. Orthologs for the *apxIICA* genes were identified in all strains but not in *A. minor* NM305^T^. In fact, an intact and a complete *apxIICABD* operon was located between the *aspC* and *folC* genes in both “*A. porcitonsillarum*” 9953L55 and *A. minor* 202, but no *apxI*, *apxIII*, or *apxIVA* genes were found, consistent with previous observations [1, 7, 30]. This *apxIICABD* operon was shown to be responsible and sufficient for RTX toxin ApxII expression and secretion and, consequently, for their hemolytic phenotype [7, 30].

Regarding the main differences in sugar utilization, orthologs encoding the *β*-galactosidase were present in all four strains accounting for their positive reaction with the o-nitrophenyl-*β*-D-galactopyranoside (ONPG) test [1, 5]. However, a full *lac* operon, i.e., including genes encoding the transcriptional regulator (*lacI*), the lactose permease (*lacY*), and the *α*-galactosidase (*melA*), was identified only in the “*A. porcitonsillarum*” and in both *A. minor* strains. The absence of the lactose permease and the *α*-galactosidase provides an explanation for the inability of *A. pleuropneumoniae* to ferment lactose and raffinose, respectively, in contrast to most *A. minor* strains [5, 27]. Consistently, we confirmed by testing lactose and raffinose fermentation that “*A. porcitonsillarum*” 9953L55 also produces acid from both sugars.

While *A. pleuropneumoniae* does not ferment trehalose, most *A. minor* strains are trehalose fermenters [5]. We identified a full *tre* operon with genes encoding the HTH transcriptional regulator (*treR*), the PTS trehalose transporter (*treP*), and the trehalose-6-phosphate hydrolase (*treA*) only in “*A. porcitonsillarum*” 9953L55 and *A. minor* NM305^T^, suggesting that both strains are able to import and ferment trehalose. As expected, when testing their ability to utilize trehalose, acid production was observed for both 9953L55 and NM305^T^, but not for S4074^T^.

On the other hand, we found no orthologs in “*A. porcitonsillarum*” 9953L55 and in both *A. minor* strains for the *mtlD* and *mtlA* genes, which code for the PTS mannitol transporter and the mannitol-1-phosphate-5-dehydrogenase in *A. pleuropneumoniae*, respectively, providing an explanation for their inability to assimilate and/or ferment mannitol [1, 5].

Most COGs shared only by “*A. porcitonsillarum*” 9953L55 and *A. minor* 202 were genes involved in different metabolic pathways, iron transport, response to stimuli, and quorum sensing. However, we also identified many orthologs for genes related to the CRISPR/Cas system, which represents the bacterial adaptive immune system against phages. Further analysis showed that both 9953L55 and 202 possess a subtype I-C CRISPR/Cas system, including a CRISPR array containing 37 repeat units in the “*A. porcitonsillarum*” strain. In contrast, complete subtypes II-C and I-F (yet in a particular genetic rearrangement) were identified in *A. minor* NM305^T^ and *A. pleuropneumoniae* S4074^T^, respectively.

Regarding phages, only one intact HP2-related *Haemophilus* prophage and an incomplete prophage region of 6.2 kb were identified in “*A. porcitonsillarum*” 9953L55 between positions 1,610,930–1,645,906 and 1,650,425–1,656,579, respectively. Interestingly, different intact *Haemophilus* as well as enterobacterial prophages were found in *A. minor* NM305^T^ and *A. pleuropneumoniae* S4074^T^, but not in *A. minor* 202 (data not shown).

Among the COGs identified exclusively in “*A. porcitonsillarum*” 9953L55 and *A. minor* NM305^T^, the IS*Apl1* was the most abundant with 10 copies present in the genome of the “*A. porcitonsillarum*” strain. This IS is typically found in *Actinobacillus* spp. and has been recently associated with the widespread of the colistin-resistance gene *mcr-1* in different genetic backgrounds [31, 32]. However, in 9953L55, the IS*Apl1* did not flank any known antibiotic resistance genes.

Nevertheless, both “*A. porcitonsillarum*” 9953L55 and *A. minor* NM305^T^ strains possessed a tetracycline resistance operon containing *tet*(B), which was located on a Tn*10* mobile element flanked by two IS*Vsa5* in opposite orientation [33]. This mobile element, which is widely disseminated among different bacterial species, was also found on integrative conjugative elements in *A. pleuropneumoniae* (ICE*Apl1*) and other *Pasteurellaceae*, such as *Haemophilus parainfluenzae* (ICE*Hpa*T3T1) [34, 35].

Regarding antimicrobial resistance, we note that in “*A. porcitonsillarum*” 9953L55, *tet*(B) was the only resistance gene identified by the *in silico* analysis with ResFinder. However, we additionally identified in this strain, as well as in *A. minor* 202, an ortholog encoding a major facilitator superfamily (MFS) transporter (LmrB) potentially associated with lincomycin resistance.

**Figure 4:**
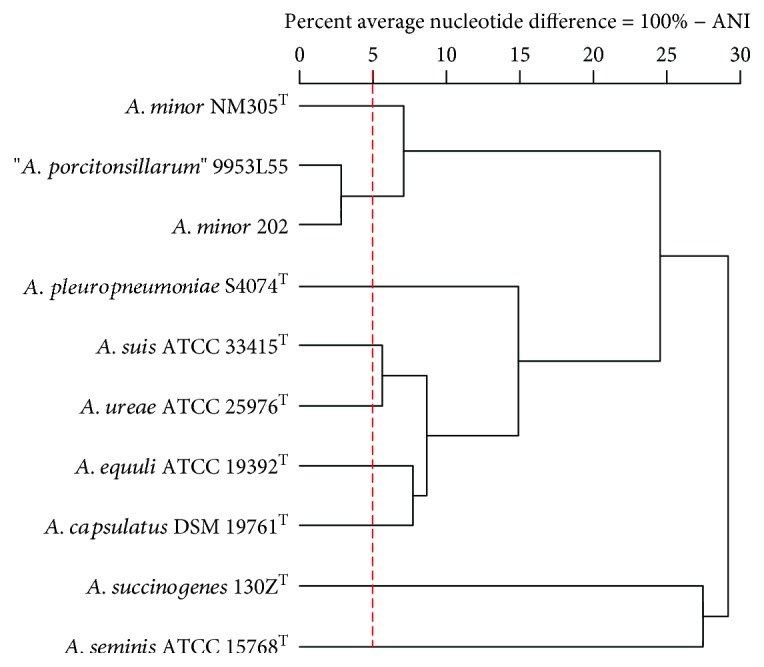
Cluster analysis of average nucleotide identity (ANI) values obtained by BLAST-based pairwise comparisons of 10 *Actinobacillus* spp. genome sequences. The distance matrix representing the ANI divergence (defined as 100% − ANI) was used for the complete linkage hierarchical clustering. The vertical dashed line represents the 95% species cutoff value.

Pairwise comparisons of the genome sequences of these four strains and the type strains of six other *Actinobacillus* spp. based on ANI confirmed the close relationship between “*A. porcitonsillarum*” and *A. minor* (Figure 4). However, ANIb values for “*A. porcitonsillarum*” 9953L55 and *A. minor* 202 were above the 95% species criteria between each other, but were below for both strains when compared with *A. minor* NM305^T^ (Suppl.  Table S1) [24, 36], indicating that 9953L55 and 202 may belong to a distinct new species closely related to *A. minor*, as suggested previously [1, 7]. Since ANIm may be more robust for genomes sharing >90% sequence similarity [37], we also implemented ANIm for pairwise comparisons of “*A. porcitonsillarum*” 9953L55 and both *A. minor* strains. The ANIm values for 9953L55 and 202 correlated well with the ANIb values; that is, both were 97.3% between each other but 93.6% when compared with NM305^T^, supporting once more the novel species hypothesis.

The same conclusions were drawn also from *is*DDH based on genome BLAST distance phylogeny, with only “*A. porcitonsillarum*” 9953L55 and *A. minor* 202 exhibiting *is*DDH values > 70%, i.e., above the same-species threshold (Suppl.  Table S2) [25]. Intriguingly, in a previous study, DNA-DNA relatedness assessed by a classic DNA-DNA hybridization (DDH) method showed borderline species-level values for 202 compared with other *A. minor* strains and, in particular, DDH values < 70% and a melting temperature difference > 5°C for 202 compared with NM305^T^, already indicating that these two strains may not belong to the same species [5, 38]. Of note, it has been suggested previously that ANI values ≥ 96% and *is*DDH values > 70% (at the upper 95% confidence interval) are good predictors of the same-species genomes in *Aeromonas* spp. [39].

## 4. Conclusions

In conclusion, we implement herein the ONT and Illumina sequencing technologies to obtain the first, complete, and highly accurate genome sequence of an “*A. porcitonsillarum*” strain and highlight its main features and differences compared to those of *A. pleuropneumoniae* and *A. minor*. Pairwise genome comparisons of 9953L55 with *A. minor* 202 and NM305^T^ based on both ANI and *is*DDH support previous observations that “*A. porcitonsillarum*” should be recognized as a new species closely related to *A. minor*, to which strain 202 also belongs. This would be essential to clearly differentiate this nonpathogenic species from the pathogenic *A. pleuropneumoniae* in diagnostic settings and, consequently, in eradication programs.

## Data Availability

The data used to support the findings of this study are available from the corresponding author upon request.

## Supplementary Materials

Supplementary Materials Table S1: the similarity matrix of the *Actinobacillus* spp. genome sequences based on average nucleotide identity (ANI) of pairwise sequence alignments performed with BLAST (ANIb). Table S2: the* is*DDH values estimated for “*A. porcitonsillarum*”, *A. minor* 202, *A. minor* NM305^T^, and *A. pleuropneumoniae* S4074^T^.
