# Supplementary material for: Comparative Genomics of the First and Complete Genome of “Actinobacillus porcitonsillarum” Supports the Novel Species Hypothesis
Source: Int J Genomics. 2018 Sep 30;2018:5261719. doi: 10.1155/2018/5261719 (PMC6186353; doi:10.1155/2018/5261719)
Supplement: Supplementary Materials — Table S1: the similarity matrix of the Actinobacillus spp. genome sequences based on average nucleotide identity (ANI) of pairwise sequence alignments performed with BLAST (ANIb). Table S2: the isDDH values estimated for “A. porcitonsillarum”, A. minor 202, A. minor NM305T, and A. pleuropneumoniae S4074T. [file 5261719.f1.pdf]

Table S1. Matrix representing the average nucleotide identity (ANI) values (%) based on BLAST pairwise sequence alignments

	A. porcitonsillarum **9953L55**	***A. minor*** **NM305T**	***A. minor*** **202**	***A. pleuropneumoniae*** **S4074T**	***A. equuli*** **19392T**	***A. succinogenes*** **130ZT**	***A. suis*** **ATCC 33415T**	***A. ureae*** **ATCC 25976T**	***A. capsulatus*** **DSM 19761T**	***A. seminis*** **ATCC 15768T**
A. porcitonsillarum **9953L55**	100	93.07	96.97	75.97	76.27	71.01	76.25	76.21	75.80	71.90
A. minor **NM305T**	92.96	100	92.93	75.73	76.10	70.57	76.16	75.90	75.80	71.31
A. minor **202**	97.16	92.94	100	75.74	76.04	70.47	76.08	75.97	75.82	71.34
A. pleuropneumoniae **S4074T**	75.81	75.85	75.77	100	86.22	71.92	86.68	85.84	85.50	72.03
A. equuli **19392T**	76.17	76.16	76.12	85.91	100	71.51	93.01	92.39	92.36	71.45
A. succinogenes **130ZT**	70.85	70.81	70.87	71.74	71.52	100	71.54	71.41	71.12	72.84
A. suis **ATCC 33415T**	76.24	76.26	76.29	86.53	92.96	71.66	100	94.25	91.73	71.58
A. ureae **ATCC 25976T**	75.90	75.70	75.89	85.58	92.26	70.97	94.41	100	91.35	71.11
A. capsulatus **DSM 19761T**	75.59	75.53	75.62	85.09	92.30	71.01	91.67	91.30	100	71.25
A. seminis **ATCC 15768T**	71.44	71.32	71.33	71.65	71.18	72.58	71.44	71.23	71.23	100

Table S2. DNA-DNA hybridization (DDH) estimate values (%) based on *in silico* DDH

	A. porcitonsillarum 9953L55	*A. minor* 202	*A. minor* NM305^T	*A. pleuropneumoniae* S4074^T
A. porcitonsillarum 9953L55	-	76.2 [73.2 – 79.0]	16.0 [13.0 - 19.5]	23.3 [21.0 - 25.8]
A. minor 202	76.2 [73.2 – 79.0]	-	52.4 [49.7 - 55.1]	22.8 [20.6 - 25.3]
A. minor NM305^T	52.6 [49.9 - 55.3]	52.4 [49.7 - 55.1]	-	16.0 [13.0 - 19.5]
A. pleuropneumoniae S4074^T	23.3 [21.0 - 25.8]	16.3 [13.3 - 19.7]	22.8 [20.6 - 25.3]	-

Note. Model-based confidence intervals are specified in square brackets
